# The Impact of Immune-Related Adverse Events on the Survival of Patients Treated with Immune Checkpoint Inhibitors: The Distinct Role of Cardiac Toxicities

**DOI:** 10.3390/jcm14217794

**Published:** 2025-11-03

**Authors:** Ileana-Raluca Pătru, Alexandra-Valentina Anghel, Eusebiu Robert Galeschi, Lorena Carolina Bătăuș, Andreea-Iuliana Ionescu, Alina Gabriela Negru, Maria Alexandra Barbu, Maria Iordache, Ionuț-Lucian Antone-Iordache

**Affiliations:** 1 Medical Oncology Department, Colțea Clinical Hospital, 030167 Bucharest, Romania; ileana-raluca.patru@drd.umfcd.ro (I.-R.P.); mariageo.iordache@gmail.com (M.I.); 2 Faculty of Medicine, “Carol Davila” University of Medicine and Pharmacy, 020021 Bucharest, Romania; alexandra-valentina.anghel@stud.umfcd.ro (A.-V.A.); eusebiu-robert.galeschi0720@stud.umfcd.ro (E.R.G.); lorena-carolina.bataus0720@stud.umfcd.ro (L.C.B.); antoneiordachelucian@stud.umfcd.ro (I.-L.A.-I.); 3 Radiotherapy Department, Colțea Clinical Hospital, 030167 Bucharest, Romania; 4 Cardiology Department, University of Medicine and Pharmacy “Victor Babes” Timisoara, 300041 Timisoara, Romania; alinanegru@umft.ro; 5 Medical Oncology Department, “Carol Davila” University of Medicine and Pharmacy, 020021 Bucharest, Romania; maria.barbu@umfcd.ro

**Keywords:** immune checkpoint inhibitors, immune-related adverse events, cardiotoxicity, survival analysis, progression-free survival, real-world data

## Abstract

**Background:** Immunotherapy with immune checkpoint inhibitors (ICIs) has fundamentally transformed cancer treatments. Unfortunately, its benefits are accompanied by the occurrence of immune-related adverse events (irAEs). While non-cardiac irAEs have been consistently associated with a favorable prognosis, the impact of cardiac toxicities remains insufficiently explored. **Methods:** We conducted a retrospective, observational study at the Oncology Department of Colțea Clinical Hospital, Bucharest. All the patients treated with ICIs between 1 May 2019 and 1 February 2024 were selected in the initial cohort. Of 512 eligible patients, 435 were included in the final analysis, with comprehensive recordings of clinical, oncological, and cardiac monitoring parameters, and at least one complete cycle of ICI treatment. Adverse events were classified according to CTCAE v5.0, and overall survival (OS) and progression-free survival (PFS) were assessed using Kaplan–Meier estimates and Cox regression models. **Results:** Our results showed that patients who developed non-cardiac irAEs experienced a significant survival benefit: median OS 26.0 months (95% CI, 15.5–NA) vs. 13.9 months (95% CI, 12.4–16.5), 0.66 (95% CI, 0.49–0.9) hazard ratio (HR); median PFS 12.3 months (95% CI, 8.1–26.0) vs. 8.7 months (95% CI, 7.3–10.3), 0.74 (95% CI, 0.56–0.97) HR. Conversely, patients with cardiac toxicities did not derive the same advantage, with similar OS and PFS values that did not reach statistical significance: median OS 15.0 months (95% CI, 13.3–19.3) vs. 15.8 months (95% CI, 12.0–30.3), 1.11 (95% CI, 0.78–1.57) HR; median PFS 9.1 months (95% CI, 7.6–10.4) vs. 8.1 months (95% CI, 5.3–19.3), 1.003 (95% CI, 0.72–1.39) HR. **Conclusions:** These findings support the role of non-cardiac irAEs as markers of favorable therapeutic response, while cardiac irAEs do not confer the same prognostic benefit. The results underscore the importance of active cardiovascular monitoring and close multidisciplinary collaboration in the management of patients receiving ICIs.

## 1. Introduction

Immunotherapy has revolutionized cancer treatment over the past two decades, providing significant survival benefits across multiple solid tumor types [1,2]. Immune checkpoint inhibitors (ICIs), such as anti-PD-1, anti-PD-L1, and anti-CTLA-4 antibodies, play a crucial role in reactivating the antitumor immune response by blocking inhibitory signals that allow tumor cells to evade immune surveillance [1,3].

However, intense stimulation of the immune system is associated with an increased risk of immune-related adverse events (irAEs), which may involve virtually any organ and range from mild, self-limiting conditions to severe, life-threatening manifestations [3,4]. The most common irAEs affect the skin, gastrointestinal tract, endocrine glands, and lungs [4,5]. In contrast, cardiac toxicities-such as myocarditis, pericarditis, or conduction abnormalities- are rare but potentially fatal, with mortality rates reported between 38% and 50% in the literature [6,7,8].

Paradoxically, several studies have demonstrated that the occurrence of non-cardiac irAEs correlates with a more effective antitumor immune response and improved overall survival, suggesting that such events may serve as an indirect marker of immune system activation [4,5,9]. Conversely, the prognostic impact of cardiac irAEs remains poorly understood, with existing data being both limited and inconsistent, particularly regarding the coexistence of cardiotoxicity and therapeutic efficacy [6,9,10].

Current cardio-oncology guidelines recommend careful monitoring of patients receiving ICIs, including the use of biomarkers and imaging for early diagnosis, as well as immediate discontinuation of treatment when myocarditis is suspected [7]. At the same time, the literature reports heterogeneous outcomes regarding the impact of cardiac toxicities on survival, ranging from neutral effects to markedly unfavorable prognoses in severe cases [8,9,10].

Against this background, our retrospective study aims to evaluate the relationship between irAE occurrence and survival in patients treated with ICIs, with a particular focus on differentiating between cardiac and non-cardiac toxicities. In addition, we assess progression-free survival (PFS) according to the adverse event profile, in order to provide an integrated perspective on the role of immunotoxicity in shaping cancer prognosis.

## 2. Materials and Methods

We conducted a retrospective observational study including patients treated with immune checkpoint inhibitors (ICIs) in the Oncology Department of Colțea Clinical Hospital, Bucharest, Romania. The analyzed cohort was derived from an internal clinical database containing information on adverse events and oncological outcomes of patients who received immunotherapy between 1 May 2019, and 1 February 2025.

**Figure 1:**
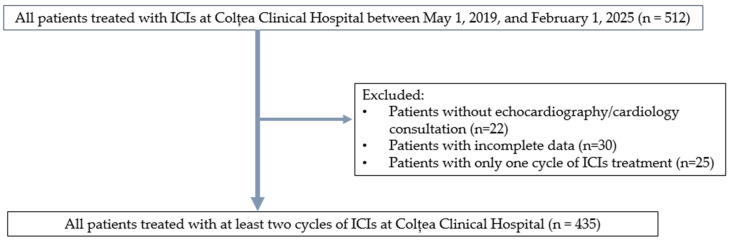
Overall study design.

Eligible patients were adults (≥18 years) diagnosed with solid tumors (including lung, renal, urothelial, melanoma, and head and neck cancers) and treated with ICIs, such as Nivolumab, Pembrolizumab, Atezolizumab, Durvalumab, or Avelumab. Exclusion criteria were the absence of data regarding disease course, survival, or adverse events; lack of basic cardiologic monitoring (at least echocardiography and electrocardiogram before and during therapy); and receipt of fewer than two cycles of immunotherapy (Figure 1).

### 2.1. Data Collection

Data were manually extracted from electronic records. The analyzed variables included: demographic and clinical data (sex, age at diagnosis, tumor type, and line of treatment), oncological treatment (type of ICI administered and treatment duration), immune-related adverse events (cardiac and non-cardiac, with severity graded according to CTCAE v5.0), survival data (vital status (alive/deceased), date of death, and disease progression status), cardiac biomarkers (troponin and CK-MB levels at the time of irAE onset, where available). Immune-related adverse events (irAEs) were categorized into cardiovascular and non-cardiovascular subgroups. Cardiac irAEs included myocarditis, pericardial disease (pericarditis or pericardial effusion), arrhythmias (atrial fibrillation, conduction disturbances), while cardiomyopathy, vascular toxicity, and hypertension were categorized under cardiovascular events. For each cardiac irAE, severity was graded according to CTCAE v5.0. An ECG was performed before every ICI administration. As per internal protocol, any new onset symptom such as dyspnea (even at minor exertion), palpitations, chest pain are investigated with an ECG and cardiac biomarkers (troponin and CK-MB). Patients are referred to a cardiologist and in select cases echocardiography is performed. A joint oncologist-cardiologist team assesses each case and decides further management.

Non-cardiovascular irAEs included dermatologic (rash, pruritus, vitiligo), gastro-intestinal (colitis, diarrhea, hepatitis), endocrine (thyroid dysfunction, hypophysitis, adrenal insufficiency), pulmonary (pneumonitis), and renal (nephritis) toxicities.

### 2.2. Statistical Analysis

JASP 0.95.1 was used. Categorical data were described as numbers and percentages, while continuous data were presented as mean and standard deviation. Overall survival (OS) and progression-free survival (PFS) were estimated using the Kaplan–Meier curve and compared with the log-rank test, dividing patients by the following predictors: presence of non-cardiac irAES, presence of cardiac irAEs, presence of severe cardiac irAEs, and presence of any cardiovascular event. The association between non-cardiac irAEs, cardiac irAEs occurrence and survival (be it overall or progression-free) was assessed using simple Cox regression analyses, the assumption of proportional hazards was checked by plotting the Schoenfeld residuals. An exploratory analysis was performed on subgroups made of only lung cancer patients and only head and neck patients. Kaplan–Meier plots and simple Cox regressions were performed to investigate if non-cardiac and cardiac irAEs were influencing the survival of the subgroups in any way. The threshold for statistical significance was set at *p* < 0.05.

## 3. Results

### 3.1. Demographic Analysis

The demographic characteristics of our population are depicted in Table 1.

The analyzed cohort included 435 patients, predominantly male, with good performance status (ECOG 0–2 in most cases). Hypertension was present in approximately half of the patients, while about 20% had diabetes and one-quarter had hypercholesterolemia. The most common diagnoses were lung cancer and head and neck cancer, followed by melanoma, renal cell carcinoma, and urothelial carcinoma. Most patients had received prior chemotherapy and/or radiotherapy. Regarding the type of ICI administered, pembrolizumab and nivolumab were the most frequently used. Overall, one-third of the patients developed cardiovascular events, and 12.6% experienced cardiac irAEs, with arrhythmias being the most frequent manifestation. Cardiovascular events are described in Table 2.

### 3.2. Survival Analysis for the Whole Population

**Figure 2:**
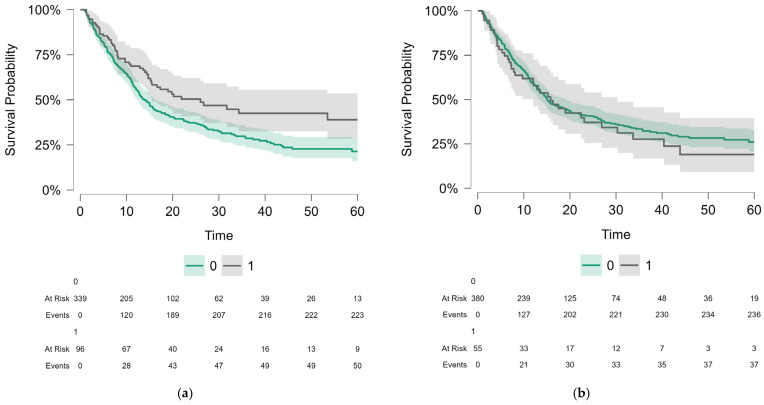
Kaplan-Meyer curves showing the following variables: (**a**) OS comparison of no non-cardiac irAEs (0) vs. non-cardiac irAEs (1) patients, p-value of 0.008. The curves showed a clear and consistent separation throughout the follow-up period, indicating a favorable prognostic effect of these events. (**b**) OS comparison of no cardiac irAEs (0) vs. cardiac irAEs patients (1), *p*-value of 0.56. Although the curves suggest a trend toward poorer prognosis in the presence of cardiac toxicities, the lack of statistical significance likely reflects both the low incidence of these events and the limitations of statistical power. These findings highlight the contrast between non-cardiac irAEs, which correlate with survival benefits, and cardiac irAEs, which remain rare but carry distinct prognostic implications.

We performed survival analysis regarding OS and PFS for the whole population, divided by the presence of cardiac events, cardiac irAEs, severe cardiac irAEs and noncardiac irAEs. Results of Kaplan–Meier survival analysis and log-rank tests were shown in Table 3. While non-cardiac irAEs significantly increased the OS and PFS, the other predictors did not influence survival in a significant way. For clarity, Figure 2 depicts the main objectives of our paper by employing a Kaplan–Meier curve along with number of patients at risk.

We also calculated the hazard ratio using the simple Cox proportional hazards models (Table 4). The proportional hazards assumption was checked by plotting the Schoenfeld residuals. The results remain consistent, with only non-cardiac irAEs being a significant predictor, showing better OS and PFS in the adverse reaction subgroup.

### 3.3. Survival Analysis for Cancer Subgroups

We chose the only populous enough subgroups (lung cancer and head and neck cancer patients) in our cohort to further investigate the effect of non-cardiac irAEs and cardiac irAEs on their OS and PFS. Results are summarized in Table 5 and Table 6. We applied the same statistical tests as on the whole population, employing Kaplan–Meier survival analysis with a log-rank test, and simple Cox proportional hazards models. In the case of lung cancer patients, cardiac irAEs were associated with a statistically significant lower OS and PFS. The rest of the results followed the general trend of the global analysis, but with lower sample sizes, there was less statistical power, and therefore could explain the not statistically significant *p*-values.

## 4. Discussion

### 4.1. General Considerations

Immunotherapy with immune checkpoint inhibitors (ICIs) has profoundly reshaped the paradigm of cancer treatment, offering patients with various malignancies significantly improved chances of durable responses and prolonged overall survival (OS). However, the same mechanism by which ICIs reactivate antitumor immunity also promotes the occurrence of immune-related adverse events (irAEs), with a broad spectrum of manifestations that can involve virtually any organ. In recent years, multiple studies have highlighted that the development of irAEs often correlates with treatment efficacy, supporting the hypothesis that autoimmunity may serve as an indirect marker of effective antitumor immune activation [5,11,12].

Our findings confirm this association for non-cardiac irAEs but also emphasize the distinctiveness of cardiovascular events. While cutaneous, endocrine, and gastrointestinal toxicities consistently correlated with a favorable prognosis, cardiac irAEs did not appear to confer a similar benefit. This differentiation has major implications both for clinical practice—where monitoring and management strategies must be tailored to the type of toxicity—and for understanding the biological mechanisms underlying immunotherapy.

Our analysis further confirms the distinct prognostic role of non-cardiac and cardiac irAEs in patients treated with ICIs. Across the entire cohort, the occurrence of non-cardiac irAEs was associated with significant benefits in both OS and progression-free survival (PFS), suggesting that these toxicities may function as indirect markers of effective antitumor immune activation. This observation is consistent with evidence from large population-based studies. For instance, Fan et al., in a meta-analysis including over 20,000 patients, demonstrated that irAEs significantly reduce the risk of progression and mortality [11], while Hussaini et al. reported hazard ratios of 0.44 for OS and 0.58 for PFS in patients with irAEs [5]. Similar findings have been reported in melanoma, non-small cell lung cancer (NSCLC), and renal cell carcinoma, where cutaneous, endocrine, and gastrointestinal irAEs have consistently been associated with effective immune stimulation and sustained antitumor responses [12].

In contrast, cardiac events associated with ICIs did not provide the same prognostic advantage. In our cohort, they appeared globally neutral but carried negative implications in specific tumor subgroups. Notably, in lung cancer patients, the presence of cardiac irAEs was associated with a significant reduction in OS, with a 59% increased risk of death. These findings are in line with international reports. Ozaki et al., as well as Gong and Braghieri, demonstrated that cardiovascular events under ICIs were associated with reduced survival in advanced NSCLC [10,13,14], while Braghieri et al. confirmed similar results in a multicenter retrospective analysis [13]. Gong and colleagues also reported unfavorable outcomes when cardiovascular toxicities occurred [15]. Conversely, Cao et al. reported more nuanced results, showing that ICI-associated myocarditis did not significantly influence OS or PFS in lung cancer, but increased cardiovascular mortality in severe cases [16]. This variability across studies likely reflects the rarity of such events (incidence < 2%) and the methodological challenges of standardizing diagnosis and reporting.

In head and neck cancer, patients with non-cardiac irAEs in our study showed a clear trend toward improved OS and PFS, although statistical significance was not reached. This finding is plausible given the relatively small number of cases in our cohort but is supported by the broader literature, where non-cardiac irAEs have been associated with higher objective response rates and prolonged survival in larger series [12,16]. Overall, these results reinforce the concept that non-cardiac irAEs constitute a robust clinical marker of favorable response to ICIs, whereas cardiac irAEs follow a different trajectory, offering no prognostic benefit and, in some contexts, such as lung cancer, potentially worsening outcomes [10,17].

### 4.2. Non-Cardiac irAEs as Prognostic Markers

In our cohort, patients who developed non-cardiac irAEs demonstrated a significant survival advantage compared with those without such events, both for OS (26 months vs. 13.9 months) and PFS (12.3 months vs. 8.7 months). This association is consistent with meta-analyses and multicenter cohorts showing that the occurrence of irAEs correlates with superior therapeutic response and a reduction in the risk of progression and death (e.g., HR 0.44 for OS and HR 0.58 for PFS) [4,5,11,18,19]. From a biological perspective, these toxicities may reflect a strong antitumor immune activation, sufficient to generate extra-tumoral manifestations, thereby explaining their value as indirect clinical biomarkers of ICI efficacy [4,5,11,18].

#### 4.2.1. Dermatologic Toxicities

Maculopapular rash, pruritus, and vitiligo are among the most common non-cardiac irAEs, mostly grade 1–2, and are easily manageable with emollients, antihistamines, and/or topical corticosteroids. Several series have reported an association between cutaneous toxicities and improved prognosis—particularly vitiligo in melanoma patients—suggesting a link between antitumor immune response and cutaneous autoimmunity [5,11,20]. In clinical practice, these events rarely require permanent discontinuation of ICIs, which allows maintenance of dose/intensity and likely contributes to survival benefit [5,20].

#### 4.2.2. Endocrine Toxicities

Thyroid dysfunction (hyper-/hypothyroidism) and hypophysitis are the most common; they frequently require long-term hormone replacement therapy (e.g., for hypothyroidism or hypocortisolism). Although they may affect quality of life, these irAEs are generally manageable and do not require long-term ICI discontinuation. In NSCLC and other solid tumors, the presence of endocrine irAEs has consistently been associated with improved OS and PFS, independent of treatment line [4,18,19]. Thus, endocrine toxicities appear to be the “safest” clinical markers of effective immune activation, with minimal impact on treatment continuity [4,18].

#### 4.2.3. Gastrointestinal and Hepatic Toxicities

Immune-mediated colitis and diarrhea show wide variability in severity; grade 3–4 events require systemic corticosteroids and, occasionally, anti-TNF agents. Although they may lead to temporary suspension of ICI therapy, multiple cohorts have observed that patients with immune colitis experience superior OS compared with those without irAEs, including in NSCLC and melanoma [19,20,21]. Immune-mediated hepatitis is less frequent but can reach high-grade severity; rapid control of liver enzymes and a clear strategy for ICI reintroduction after resolution remain essential to preserve therapeutic benefit [21].

#### 4.2.4. Pulmonary Toxicities

Immune-mediated pneumonitis is less frequent than cutaneous or endocrine toxicities, but it remains one of the most serious non-cardiac irAEs, with risk of respiratory failure and mortality, especially in grade ≥ 3 cases. Despite its severity, some studies did not identify a clear positive relationship between pneumonitis and OS, suggesting that the prognostic benefit of “immune activation signaling” may be counterbalanced by the clinical impact of pulmonary events [22]. This asymmetry reinforces the idea that “not all non-cardiac irAEs are equal” in terms of prognosis: events that are easily/moderately manageable (cutaneous, endocrine) are more often associated with better OS/PFS, whereas severe toxicities (pneumonitis) may diminish survival benefit [5,11,19,20,22].

#### 4.2.5. Renal Toxicities (Nephritis)

Although less frequent than cutaneous or endocrine events, immune-mediated nephritis is reported in large series and requires careful monitoring of renal function. It generally responds to corticosteroid therapy, and ICI rechallenge can be considered in selected cases after resolution. From a survival perspective, nephritis has not shown as consistent a favorable signal as cutaneous/endocrine toxicities, but the absence of a clear negative effect differentiates it from cardiovascular events [4,5,11,18].

Non-cardiac irAEs often occur within the first 6–12 weeks (dermatologic/endocrine), but they may also present late, sometimes after months of therapy. Although high-grade (≥3) events are associated with treatment interruptions and may reduce survival advantage, most non-cardiac events are low grade and therefore compatible with treatment continuation or brief interruptions followed by rechallenge [4,5,11,18,19,20,21]. This “therapeutic compatibility” may explain the difference compared with cardiac irAEs, where definitive discontinuations are more frequent.

The association between irAEs and survival may be influenced by temporal biases (e.g., immortal-time/guarantee-time). Many analyses attempt to mitigate these effects through landmark analysis or time-dependent variables; even so, the favorable signal for non-cardiac irAEs remains consistent across meta-analyses and cohorts [4,5,11,18,19]. In interpretation, corticosteroid/immunosuppressive exposure should also be considered: generally, early intervention and adequate dosing for the management of non-cardiac irAEs do not appear to completely negate ICI benefit, particularly when rechallenge is possible after resolution [19,20,21].

From a practical perspective, these results support active monitoring for early detection of non-cardiac irAEs, standardized management protocols (CTCAE v5.0), and a clear strategy for rechallenge after resolution in appropriate cases. Especially for cutaneous and endocrine toxicities, treatment continuity appears essential to maintain survival benefit, in line with international data [4,5,11,18,19,20].

Non-cardiac irAEs remain a heterogeneous group, but overall, they represent a favorable clinical marker of response to ICI therapy, with the strongest positive signal observed in cutaneous and endocrine events; in contrast, pneumonitis and high-grade GI/hepatic events require a delicate balance between toxicity management and maintenance of efficacy [4,5,11,18,19,20,21,22].

### 4.3. Cardiac irAEs: Neutral or Negative Impact

Our results showed that patients with cardiac irAEs had a lower median overall survival (OS) (11.4 months vs. 17.6 months) and a shorter median progression-free survival (PFS) (4.3 months vs. 5.8 months), although the differences did not reach statistical significance. These findings suggest that cardiac toxicities do not confer the same prognostic advantage as non-cardiac ones and, in certain situations, may even exert a negative impact.

In our cohort, we identified a broad spectrum of cardiovascular irAEs, the most frequent being myocarditis, pericardial involvement, and arrhythmias.

Myocarditis had an incidence of approximately 3% and was predominantly grade 2–3. Although rare, ICI-induced myocarditis is one of the most severe complications, with mortality rates reported between 40–50% in severe cases, especially when diagnosis is delayed [23,24,25]. Moreover, its typically early onset (within the first 30–35 days after immunotherapy initiation) differentiates it from many other irAEs, which often occur later. The risk is higher in patients receiving combination therapy with PD-1/PD-L1 and CTLA-4 inhibitors compared with monotherapy [24]. In international studies, myocarditis was frequently diagnosed based on elevated troponin, ECG changes, and cardiac MRI findings; however, heterogeneity in diagnostic criteria may explain the variability in reported incidence.

Pericardial involvement was observed in approximately 3.4% of our patients, ranging from minor effusions to severe pericarditis, often requiring immunotherapy discontinuation. Data from international cohorts confirm this spectrum: in a TriNetX-based analysis (~88,900 patients), the incidence of pericarditis was 0.22%, cardiac tamponade 0.47%, and pericardial effusion up to 4.7%, all associated with increased one-year mortality [26]. Gong et al. further demonstrated that patients with pericardial events under ICI had a more than fourfold increased risk of death compared with those without, confirming their unfavorable prognostic impact [14,27].

Arrhythmias were identified in ≈8% of patients, including atrial fibrillation, supraventricular tachyarrhythmias, and conduction disturbances. These values are at the upper limit of those reported internationally (4–5% for atrial fibrillation) [28,29]. Arrhythmias associated with ICI may result from diffuse myocardial inflammation or conduction system injury, and in cases of myocarditis, they may include life-threatening ventricular arrhythmias [30]. Pharmacovigilance studies have reported an increasing incidence of atrial fibrillation, particularly with PD-1/PD-L1 inhibitors and with the ipilimumab–nivolumab combination, where the risk is significantly higher compared with monotherapy [31].

Cardiomyopathy and vascular toxicity were rare in our cohort but should be emphasized, as they may directly impact prognosis by reducing ejection fraction and through acute vascular complications (e.g., severe hypertension, thromboembolic events). Although their incidence is low, their clinical impact is important, often requiring hospitalization and, in some cases, permanent discontinuation of immunotherapy [32,33].

Our findings are in line with global data: myocarditis and arrhythmias remain the most commonly reported cardiovascular irAEs, although rare, while pericardial involvement, though less frequent, is associated with poor prognosis [14,23,24,25,26,27,28,29]. Differences in incidence may reflect diagnostic criteria, reporting methods, or cohort characteristics (tumor types, ICI regimens used). Pharmacovigilance analyses suggest that these events, although rare, are likely underreported, which could explain discrepancies between centers [25,31].

Proposed mechanisms for cardiac irAEs include infiltration of autoreactive T lymphocytes, recognition of shared antigens between myocardium and tumor cells, and loss of the protective role of PD-L1 expression on cardiomyocytes, leading to uncontrolled inflammation [32]. This pathophysiological specificity explains the disproportionate severity of cardiac toxicities compared with their relatively low incidence.

From a clinical perspective, unlike non-cardiac irAEs, where many events are manageable and compatible with therapy continuation, cardiac irAEs are more frequently associated with permanent ICI discontinuation. Cardio-oncology guidelines recommend rapid evaluation with biomarkers (troponin, BNP), echocardiography, and cardiac MRI in suspected myocarditis, as well as early initiation of high-dose corticosteroids [26,30]. Moreover, close interdisciplinary collaboration between oncologists and cardiologists is essential for early diagnosis, risk stratification, and decisions regarding potential ICI rechallenge.

Our data confirm the distinct profile of cardiac irAEs: rare events, but with disproportionate severity and an unfavorable prognostic impact. While patients with non-cardiac irAEs benefit from longer survival, those with cardiac irAEs did not show the same advantage, justifying intensive cardiovascular monitoring and the implementation of standardized management protocols.

### 4.4. Mechanisms of ICI-Associated Cardiotoxicity

Unlike non-cardiac irAEs, cardiac irAEs often reflect direct immune aggression against a vital organ. Experimental data indicate that PD-1/PD-L1 signaling plays a protective role in the heart. Dual PD-1/CTLA-4 blockade causes an increased rate of cardiotoxicities [34,35]. While the mechanisms of ICI cardiotoxicity are not completely understood, several theories have emerged: cross-reactivity between myocardial cells and tumor cells, tumor cell death releasing intracellular antigens, activation of dormant self-reacting T-cell clones and overexpressed PD-L1 in ischemic myocardium all leading to T-cell infiltration coupled with direct cytotoxic injury and increased levels of pro-inflammatory cytokines [36,37]. Elevated levels of such cytokines have been linked to chronic heart failure, infarction, atherosclerosis, a recent ACC statement has reevaluated inflammation as a primary driver in the context of cardiovascular disease [37,38].

The interplay between these mechanisms of pre-existing inflammation and increased myocardial PD-L1 expression (blocked by ICI therapy) [37] may lead to patients who have pre-existing cardiovascular conditions being more likely to develop cardiac irAEs as found in a review by Wang et al. [39]. Patients being already fragile at the onset of cardiac irAEs and the high level of mortality resulting from this category of adverse events may offset any clinical benefit on survival that cardiac irAEs possess [40].

### 4.5. Clinical Implications

These results have important clinical consequences. ASCO guidelines recommend immediate discontinuation of ICIs upon suspected myocarditis, regardless of severity [41]. Cardio-oncology consensus statements stress multidisciplinary collaboration [7]. Early use of biomarkers (troponin, BNP) and imaging (echocardiography, cardiac MRI) facilitates timely diagnosis [27,42]. Rechallenge may be feasible in selected cases, though recurrence is common [43].

### 4.6. Relationship Between PFS and OS

OS remains the gold standard for immunotherapy trials, while PFS is often used as a surrogate endpoint. However, PFS does not always predict OS, particularly in immunotherapy only trials [44]. In the case of cardiac irAEs, the lack of significant PFS differences does not preclude a clinically meaningful OS impact through direct mortality or treatment discontinuation.

### 4.7. Study Limitations

This study has several strengths, including the analysis of a real-world cohort treated in a reference oncology center, the assessment of both cardiac and non-cardiac toxicities, and the integration of detailed survival data. However, some methodological limitations must be acknowledged. The retrospective design entails a potential risk of selection bias and may lead to underreporting of mild immune-related adverse events. The absence of specific immunological biomarkers and the lack of standardized imaging evaluations for all cases of cardiac toxicity limit diagnostic accuracy. Furthermore, the relatively small size of the subgroup with cardiac events reduces the statistical power of comparative analyses.

## 5. Conclusions

Our retrospective real-world study of patients treated with immune checkpoint inhibitors (ICIs) highlights the distinct prognostic roles of immune-related adverse events (irAEs). The occurrence of non-cardiac irAEs was consistently associated with a significant improvement in both overall survival (OS) and progression-free survival (PFS), supporting the hypothesis that these events may serve as indirect clinical markers of effective immune activation and favorable therapeutic response.

In contrast, cardiac irAEs did not confer the same prognostic benefit. Although rare, they were associated with a potentially unfavorable impact on OS and PFS. The lack of statistical significance likely reflects the low incidence and limited sample size of the cardiac subgroup. However, the findings are consistent with the literature, which identifies myocarditis, arrhythmias, and pericardial involvement as life-threatening complications requiring early recognition and specialized care.

From a clinical perspective, our data emphasize the importance of active monitoring in patients receiving ICIs, while the implementation of standardized cardio-oncology protocols and close collaboration between oncologists and cardiologists becomes essential for reducing associated mortality and optimizing treatment continuity.

Furthermore, these findings highlight the necessity of prospective, multicenter studies, including national registries and integrated analyses of immunological and cardiovascular biomarkers. Such efforts are crucial to fully understand the impact of cardiac irAEs and to develop tailored strategies for prevention, diagnosis, and management.

In conclusion, non-cardiac irAEs emerge as favorable prognostic markers and indicators of effective immunotherapy, whereas cardiac irAEs define a distinct and vulnerable subset of patients who require intensive cardio-oncological surveillance. This differentiation has major implications for clinical practice and represents an important step toward the individualization of immunotherapy management.

## Figures and Tables

**Table 1:** Demographic and clinical characteristics of the patients included in the study.

Variable		Number	Frequency (%)
Sex	Male	313	72.0
	Female	122	28.0
ECOG	ECOG 0	137	31.5
	ECOG 1	124	28.5
	ECOG 2	167	38.4
	ECOG 3	7	1.6
Smoking status *	Smoker	234	53.9
	No smoker	200	46.1
DM prior to ICIs	Yes	85	19.5
	No	350	80.5
HT prior to ICIs	Yes	218	50.1
	No	217	49.9
Hypercholesterolemia prior to ICIs	Yes	96	22.1
	No	339	77.9
Diagnosis	Lung cancer	219	50.3
	Head and neck cancer	137	31.5
	Melanoma	43	9.9
	Renal cell carcinoma	16	3.7
	Urothelial carcinoma	12	2.8
	Hepatocellular carcinoma	4	0.9
	Breast cancer	2	0.5
	Cancer of Unknown Primary	2	0.5
CHT prior to ICIs **	Yes	184	42.3
	No	247	56.8
RT prior to ICIs	Yes	236	54.3
	No	199	45.7
ICIs	Atezolizumab	20	4.6
	Avelumab	7	1.6
	Durvalumab	12	2.8
	Nivolumab	140	32.2
	Nivolumab + Ipilimumab	22	5.1
	Pembrolizumab	234	53.8

* One patient has missing data. ** 4 patients have missing data on previous chemotherapy. CHT—systemic chemotherapy, DM—diabetes mellitus, HT—hypertension, RT—radiation therapy.

**Table 2:** Cardiovascular events of the patients included in the study.

Cardiovascular events (n = 144, 33.1%)	Cardiomyopathy	45	10.3
	Hypertension	20	4.6
	Vascular toxicity (thrombosis)	55	12.6
Cardiac irAEs (n = 55, 12.6%)	Arrhythmias	35	8
	Grade 2	19	4.4
	Grade 3	5	1.1
	Grade 1	10	2.3
	Grade 4	1	0.2
	Pericardial disease	15	3.4
	Grade 2	6	1.4
	Grade 3	6	1.4
	Grade 1	2	0.5
	Grade 4	1	0.2
	Myocarditis	13	3
	Grade 2	8	1.8
	Grade 3	4	0.9
	Grade 4	1	0.2

**Table 3:** Analysis of overall survival (OS) and progression-free survival (PFS) for the whole population.

**OS**					
	**Number**	**RMST**	**Median**	**95% Confidence Interval**	**Log-Rank Test *p*-Value**
Global	N = 435	26.18	15.2	13.5–19.0	-
Cardiac irAEs	No N = 380	26.51	15.0	13.3–19.3	0.56
	Yes N = 55	23.91	15.8	12.0–30.3	
Non-cardiac irAEs	No N = 339	24.39	13.9	12.4–16.5	0.008
	Yes N = 96	32.45	26.0	15.5–NA	
Severe cardiac irAEs	No N = 417	26.3	15.1	13.4–19.0	0.76
	Yes N = 18	24.22	17.1	7.3–NA	
Cardiac events other than irAEs	No N = 291	25.6	14.4	12.4–17.5	0.44
	Yes N = 144	27.42	20.1	14.4–27.4	
**PFS**					
	**Number**	**RMST**	**Median**	**95% Confidence Interval**	**Log-Rank Test *p*-Value**
Global	N = 435	19.25	9.0	7.6–10.5	-
Cardiac irAEs	No N = 380	19.31	9.1	7.6–10.4	0.97
	Yes N = 55	18.69	8.1	5.3–19.3	
Non-cardiac irAEs	No N = 339	17.83	8.7	7.3–10.3	0.029
	Yes N = 96	24.25	12.3	8.1–26.0	
Severe cardiac irAEs	No N = 417	19.34	9.0	7.6–10.5	0.87
	Yes N = 18	14.19	9.2	4.6–NA	
Cardiac events other than irAEs	No N = 291	18.54	8.1	7.1–9.7	0.2
	Yes N = 144	27.42	20.1	14.4–27.4	

**Table 4:** Cox simple regressions exploring the association between irAEs and survival (OS and PFS).

Variable		Hazard Ratio	*p*-Value
OS	Non-cardiac irAEs (yes)	0.66 (0.49 to 0.9)	0.008
	Cardiac irAEs (yes)	1.11 (0.78 to 1.57)	0.56
PFS	Non-cardiac irAEs (yes)	0.74 (0.56 to 0.97)	0.029
	Cardiac irAEs (yes)	1.003 (0.72 to 1.39)	0.99

**Table 5:** Analysis of overall survival (OS) and progression-free survival (PFS) in patients with lung cancer and head and neck cancer, according to irAEs.

**OS**						
		**Number**	**RMST**	**Median**	**95% Confidence Interval**	**Log-Rank Test *p*-Value**
Lung	Global	N = 219	25.92	15.1	13.1–19.8	-
	Cardiac irAEs	No N = 181	27.35	16.6	13.7–26.0	0.025
		Yes N = 38	18.65	12.0	7.2–19.0	
	Non-cardiac irAEs	No N = 160	25.72	14.4	12.1–23.5	0.73
		Yes N = 59	26.43	16.6	14.0–34.3	
HN	Global	N = 137	21.30	11.4	9.3–15.5	-
	Cardiac irAEs	No N = 130	There were less than 10 patients with cardiac irAEs			
		Yes N = 7				
	Non-cardiac irAEs	No N = 117	18.9	11.3	8.7–14.4	0.051
		Yes N = 20	32.22	31.7	8.2–NA	
**PFS**						
		**Number**	**RMST**	**Median**	**95% Confidence Interval**	**Log-Rank Test *p*-Value**
Lung	Global	N = 219	18.92	9.3	7.4–11.1	-
	Cardiac irAEs	No N = 181	19.87	9.7	8.0–11.3	0.83
		Yes N = 38	14.30	5.9	4.3–12.0	
	Non-cardiac irAEs	No N = 160	18.43	9.3	7.5–11.2	0.76
		Yes N = 59	20.06	9.0	5.8–21.9	
HN	Global	N = 137	16.07	6.6	5.0–8.7	-
	Cardiac irAEs	No N = 130	There were less than 10 patients with cardiac irAEs			
		Yes N = 7				
	Non-cardiac irAEs	No N = 117	13.22	5.9	5.0–8.7	0.055
		Yes N = 20	26.78	8.2	4.5–NA	

HN—head and neck.

**Table 6:** Cox models simple regressions exploring the association between irAEs and survival in lung cancer and head and neck cancer subgroups.

Variable			Hazard Ratio	*p*-Value
OS	Lung	Non-cardiac irAEs (yes)	0.94 (0.64 to 1.36)	0.73
		Cardiac irAEs (yes)	1.59 (1.06 to 2.4)	0.026
	HN	Non-cardiac irAEs (yes)	0.52 (0.27 to 1.01)	0.55
PFS	Lung	Non-cardiac irAEs (yes)	0.94 (0.67 to 1.34)	0.75
		Cardiac irAEs (yes)	1.41 (0.95 to 2.09)	0.08
	HN	Non-cardiac irAEs (yes)	0.55 (0.3 to 1.02)	0.58

HN—head and neck.

## Data Availability

Data available on request due to ethical restrictions. The data presented in this study are available on request from the corresponding author and the Coltea Clinical Hospital (secretariat@coltea.ro). The data are not publicly available due to the policy of Coltea Clinical Hospital to have the approval of the Ethics Commitee for each new research study.
